# Supplementary material for: Pre- and post-operative psychological interventions to prevent pain and fatigue after breast cancer surgery (PREVENT): Protocol for a randomized controlled trial
Source: PLoS One. 2022 Jul 8;17(7):e0268606. doi: 10.1371/journal.pone.0268606 (PMC9269362; doi:10.1371/journal.pone.0268606)
Supplement: S1 File — The written confirmation that the study protocol has received an approval from the Regional Ethical Committee of South-Eastern Norway. (PDF) [file pone.0268606.s002.pdf]

Pre- and post-operative psychological interventions to prevent pain and fatigue after breast cancer surgery: a randomized controlled trial

Silje E. Reme[1,2] s.e.reme@psykologi.uio.no

Henrik. B. Jacobsen[1,2] h.b.jacobsen@psykologi.uio.no

[1]Department of Psychology, Faculty of Social Sciences, University of Oslo, Norway

[2]Department of Pain Management and Research, Oslo University Hospital, Norway

[*]Corresponding author: Silje Endresen Reme

Abstract

Background: Breast cancer is the most common cancer type among women with >1million new cases worldwide. More than 40% of them will struggle with pain and fatigue years after surgery, regardless of surgical procedure. These consequences are detrimental and result in distress and disability, including work disability. Few previous attempts have been made to prevent persistent pain and fatigue after surgery by applying a psychological approach, despite psychological risk factors being crucial in the development of both persistent pain and fatigue. In this study, we aim to develop and test an easily implementable strategy of preventing persistent pain and fatigue after breast cancer surgery. The intervention strategy involves a pre-operative hypnosis session and a web-based post-operative Acceptance and Commitment Therapy. The hypnosis was previously found effective in alleviating acute post-operative pain and fatigue in breast cancer patients, while ACT may be particularly well suited to cancer populations as it offers a model of healthy adaptation to difficult circumstances. Together they form an intervention strategy with both a preventive and a rehabilitative focus.

Methods/design: The study will be run as a randomized controlled trial where effects of the pre- and post- operative interventions will be compared to attentional control and treatment as usual (TAU). Patients will be randomized to receive either a brief hypnosis session before surgery, and a web-based psychological intervention after surgery (ACT), or mindfulness meditation administered through a mobile app before surgery and TAU after surgery. Measurement, in the form of self-reported data and biomarker data will be assessed pre-surgery, post-surgery and 3 and 6 months after surgery.

Discussion: This study will be the first study to combine a pre-operative hypnosis with a post-operative ACT to prevent pain and fatigue after breast cancer surgery. The results from our study might i) help the large group of women affected by chronic pain and fatigue after breast

cancer surgery, ii) shed light on the mechanisms involved in chronic pain development, and iii) serve as a model for other surgical procedures.

Trial registration: The project will be registered in a relevant international trial register.

Keywords: Breast cancer; pain; post-surgical pain; fatigue; hypnosis; acceptance and commitment therapy

Background

Breast cancer is the most common cancer type among women, with >1 million new cases worldwide every year (1). Survival rates for breast cancer is increasing (2), but for many patients survivorship is also characterized by disabling psychological and physical late effects (3-5). Chronic post-surgical pain (CPSP) and persistent fatigue have the most profound prevalence. CPSP has prevalence rates ranging from 25-60% depending on definition, measurement and treatment (6). In a recent Norwegian study, more than 40% of women treated for breast cancer continued to have pain 2-6 years after surgery (7), while over a third of the women in a large Danish study reported persistent pain 5-7 years after surgery (8).

Persistent fatigue is the most common symptom associated with cancer and its treatments (9). Still, studies on the occurrence of and predictors of fatigue after surgery are scarce. One of the few studies investigating persistent fatigue after surgery reported high levels of fatigue in the first two months after surgery, followed by mild-to-moderate levels of fatigue that persisted 12 months after surgery (10). In another post-operative study, a third of the women struggled with persistent fatigue up to 10 years into survivorship (11)

The pathophysiology of CPSP is still unclear. Presumably, the nature of most CPSP is neuropathic since surgery (e.g. mastectomy) may involve major nerve damage, and is associated with the highest incidence of such pain (12, 13). However, many patients with CPSP do not show any signs of neuropathic pain, or any sensory changes (3). It is thus assumed that the distinct pathophysiology reflects both peripheral and central sensitization as well as humoral factors contributing to pain. Further, the etiology of chronic pain is grounded in the bio-psycho-social model, where psychological and social factors contribute to the

development of CPSP (14). The pathophysiology of post-surgical fatigue is also largely unclear (15). The underlying mechanisms likely vary from patient to patient, with a mixture of biological and psychological underpinnings (16). Interestingly, there are several shared risk factors between pain and fatigue after surgery, including depression and outcome expectancies (17), implying that the very same processes might influence both post-surgical pain and fatigue.

Unmodifiable risk factors for CPSP involve female gender and younger age (12), as well as preoperative pain conditions and use of opioids (12, 13, 18, 19) . However, a preventive intervention could target psychological factors, which are both modifiable, robust and potent predictors of CPSP and fatigue (14, 20). The most frequent tap into anxiety, such as hypervigilance and pain catastrophizing (20-24). Other strong predictors involve depression, stress, and optimism (10, 14, 23).

Despite strong evidence supporting the role of psychological factors in the development of CPSP and fatigue, hardly any studies have investigated psychological and behavioral interventions aimed at preventing these symptoms. CPSP and fatigue *management*, however, has been the aim of several psychological and behavioral interventions. Mindfulness-based cognitive therapy on persistent pain after breast cancer surgery was recently found effective in reducing pain intensity (25), and a study led by a member of our project group found beneficial effects of CBT plus hypnosis to control fatigue in patients undergoing radiotherapy for breast cancer (26). Several studies of similar psychological interventions exist, but with limited effects (27, 28), and with no preventive focus (29-31).

Hypnosis is a non-pharmacotherapeutic technique that holds promise as a harmless and effective pre-operative intervention to alleviate acute post-surgical pain and fatigue (32, 33). Clinical research with at least 20 different surgical populations has indicated that hypnosis can reduce the need for medication, reduce post-surgical symptoms, and enhance recovery (33). Furthermore, meta-analyses (34), narrative reviews (35, 36), and randomized clinical studies (37-40) all support the potential clinical utility of hypnosis with surgical breast cancer patients. A rigorous trial from 2007 demonstrated particularly large effects of a specific 15-minute pre-operative hypnosis on analgesic needs, pain intensity and fatigue after breast cancer surgery (41). However, the results from this trial have not been replicated, and results from hypnosis trials in general lack long-term follow-up.

Acceptance and Commitment Therapy (ACT) is an evidence-based treatment for persistent pain (42, 43) and is also a good fit for treating persistent fatigue (44). ACT is well suited to cancer populations as it offers a model of healthy adaptation to difficult circumstances and has shown promise in the treatment of opioid misuse (45). There have been several calls to expand the scope of ACT interventions to the treatment of CPSP (46). In a recent initiative, ACT was applied as treatment for CPSP in The Toronto General Hospital, with preliminary promising results. Those receiving ACT demonstrated greater reductions in opioid use and pain interference, as well as reductions in depressed mood, compared to those who received treatment as usual (follow-up form medical doctor) (47, 48). In addition to being an evidence-based treatment for pain and fatigue, ACT is also among the very few psychological treatments with documented effects on return-to-work (49). Moreover, in a Norwegian study from members of the project group, a miniscule ACT follow-up intervention consisting of 1-6 phone calls, eased patients transition into the workplace (50)

Despite the fact that both CPSP and persistent fatigue are common consequences of breast cancer surgery (7, 15), with disabling effects on recovery outcomes and quality of life, hardly any studies have looked at preventive interventions from a psychological perspective. As psychological risk factors are vital, and the pathophysiology of both conditions largely unknown, interventions focusing on the psychological factors are largely needed.

Methods/Design

Aims and objectives

The primary aim of the study is to develop and test an easily implementable strategy of preventing persistent pain and fatigue after breast cancer surgery. The intervention strategy involves a pre-operative hypnosis session and a post-operative, internet-based Acceptance and Commitment Therapy (iACT). The hypnosis was previously found effective in alleviating acute post-operative pain and fatigue in breast cancer patients, while ACT may be particularly well suited to cancer populations as it offers a model of healthy adaptation to difficult circumstances. Together they form an intervention strategy with both a preventive and a rehabilitative focus.

We have the following hypotheses:

1) Hypnosis is more effective than one-session mindfulness in relieving acute post-surgical pain and fatigue

2) Hypnosis alters markers of bodily stress responses and impacts immune reactivity associated with pain and fatigue.

3) Hypnosis + iACT results in less post-surgical pain and fatigue and fewer medical prescriptions for pain medication 3 months after surgery compared to one-session mindfulness + TAU

4) Hypnosis + iACT is favorable in a cost-utility analysis, and will result in fewer days of sick leave at 1 year follow-up compared to one-session mindfulness + TAU

5) Hypnosis + iACT results in less psychological distress and fewer medical prescriptions for psychotropic medications at the 3 and 6-month follow-ups compared to one-session mindfulness + TAU

Design

The study design involves a sequential, multiple assignment, randomized trial (SMART), where participants are randomly assigned to a treatment option at two stages, both pre- and post-surgery. By randomizing participants multiple times, we can assess the effectiveness of both interventions at each stage. We will thus be able to assess the short and long-term effectiveness of the pre-operative hypnosis, both alone and in combination with the post-operative iACT. Similarly, we will be able to assess the effectiveness of the post-operative iACT, both alone and in combination with the pre-operative hypnosis.

Outcome Measures

To assess hypothesis 1, subjective experiences of pain intensity, pain unpleasantness, and fatigue will be assessed using the Numerical Rating Scale (NRS) and Visual Analogue Scales (VAS) after surgery, upon discharge. Both intensity (i.e., amount) and unpleasantness of pain will be assessed to capture its sensory and affective dimensions. Two 100-mm visual analog scales (VASs) will be used to assess fatigue and muscle weakness, in line with previous breast cancer trials of post-surgical fatigue (26, 41).

To assess hypothesis 2, we will measure cortisol in hair as a marker of hypothalamus-pituitary-adrenal (HPA) axis activity, and two measures of inflammation – high sensitive CRP

(hs-CRP) and lipopolysaccharide (LPS) reactivity. We consider hs-CRP an acceptable initial marker of systemic inflammation, whilst LPS will provide more nuanced and detailed results of immunological reactivity.

To assess hypothesis 3, NRS will be used to measure post-surgical pain, and FACIT-F (51) to measure post-surgical fatigue at 3 months follow-up. Registry data on pain-related prescriptions will further be extracted from The Norwegian Prescription Database (NorPD).

To assess hypothesis 4, we will use registry data to estimate the number of days participants in each group have been on sick leave (or other welfare benefits) during the year following surgery.

To assess hypothesis 5, we will measure psychological distress by the Hospital Anxiety and Depression Scale (52), as well as data on psychotropic prescriptions from The Norwegian Prescription Database (NorPD).

The Numeric Rating Scale (NRS):

The NRS is a discontinuous, self-report measure. It consists of a single item, in which the respondent is asked to rate the intensity of pain. This rate is done on an 11-point scale ranging from 0-10, anchored by verbal descriptors at either end of the scale. In the context of pain, the NRS has been proven to have good reliability and validity (Hawker, Mian, Kendzerska, & French, 2011; Johnson, 2005). It is a valid measure of pain intensity (0-10) that is less influenced by present mood state (54), and has been widely used to assess post-surgical pain in previous studies of women with breast cancer (3, 7, 8, 55).

The Visual Analog Scale (VAS):

The VAS is a continuous, self-report measure comprised of either a horizontal or vertical line measured at exactly 100 mm. The line is anchored by verbal descriptors, and the respondent is asked to rate the intensity of their symptom by making a mark on the line. The distance between the end of the line, representing absence of the symptom, and the mark will be measured and a score ranging from 0 to 100 will be recorded. Similar to the NRS, the VAS can be used on a variety of symptoms and will here be used to assess fatigue and muscle weakness (Hawker et al., 2011; Johnson, 2005).

The Surgical Fear Questionnaire (SFQ):

The SFQ is a reliable and valid self-report instrument designed to assess fear of surgery, and it consists of 8 items which is scored on an 11-point scale ranging from 0 (not at all afraid) to 10 (very afraid). The items target different fears concerning surgery, namely fear of operation, anaesthesia, postoperative pain, side effects, health deterioration, failed operation, incomplete recovery and long duration of rehabilitation. The structure of the SFQ can best be described by a two-factor model, in which two subscales can be distinguished: fear of immediate consequences of surgery and fear of the long-term consequences (Theunissen et al., 2014).

Life Orientation Test – Revised (LOT-R)

The LOT-R is a 10 item self-report test meant to assess dispositional optimism. The respondents are asked to rate their agreement to various statements on a 5-point scale ranging from 0 (strongly disagree) to 4 (strongly agree). Three of the items assess optimism, three assess pessimism, and the remaining four are filler items that are not meant to be included in the scoring (Hinz et al., 2017).

Hospital Anxiety and Depression Scale (HADS)

The HADS is a scale specifically designed for patients with physical illness, and it makes no references to somatic symptoms such as fatigue, insomnia or headache. It is a self-administered rating scale comprised of 14 items that are equally divided into two subscales: depression and anxiety. Each subscale therefore consists of seven items, and the respondents are asked to respond on a 4-point scale ranging from 0 to 3. Consequently, each subscale has a possible score of 0-21, and a score of 11 or higher often indicates problematic presence of either depressive or anxious symptoms (Bjelland, Dahl, Haug, & Neckelmann, 2002; Carroll, Kathol, Noyes, Wald, & Clamon, 1993; Snaith, 2003; Zigmond & Snaith, 1983).

The Pain Catastrophizing Scale (PCS)

The PCS is a self-report measure meant to assess catastrophizing. It is comprised of 13 items, each rated on a 5-point scale ranging from 0 to 4 (A. Osman et al., 2000; Augustine Osman et al., 1997; Sullivan, Bishop, & Pivik, 1995).

The 13-item Functional Assessment of Chronic Illness Therapy-Fatigue subscale (FACIT-F)

The FACIT-F is a unidimensional self-report scale meant to assess fatigue and its impact on daily life. Consisting of 13 items, the scale asks the respondents to rate their level of symptom intensity on a 5-point scale ranging from 0 to 4 (Butt et al., 2013; Yellen, Cella, Webster, Blendowski, & Kaplan, 1997). FACIT-F is a widely used measure of fatigue in breast cancer trials. It has demonstrated excellent internal consistency, high validity, and sensitivity to pick up change in patients with breast cancer (51).

Insomnia Severity Index (ISI)

The ISI is a 7-item instrument, measuring severity, nature and impact of insomnia symptoms. It is a brief screening and outcome measure for use in treatment research, and the scale corresponds in part to DSM-IV criteria for insomnia. The respondents are asked to rate their symptoms on a 5-pont scale ranging from 0 to 4, which assess the subject's current perception of symptom severity, distress and impairment (Morin, Belleville, Bélanger, & Ivers, 2011; Smith & Wegener, 2003).

The European Organization for Research and Treatment-QOL questionnaire for breast cancer specific module (EORTC QLQ-BR23)

The EORTC QLQ-BR23 is a health-related quality of life measure specifically designed for women with breast cancer. It consists of 23 items, each asking the respondent to indicate the extent to which they have experienced certain symptoms or problems on a 4-point scale ranging from "not at all" to "very much" (Nguyen et al., 2015; Sprangers et al., 1996). This measure was specifically included upon recommendation from our user representative.

Injustice Experience Questionnaire (IEQ)

The IEQ is a 12-item measure meant to assess the respondent's experience of injustice. Injustice encompasses the degree of blame as well as the magnitude and irreparability of loss related to their health condition. The items therefore consists of thoughts and feelings related to injustice. Respondents are asked to rate their experience of injustice on a 5-point scale ranging from "not at all" to "all the time" (Sullivan et al., 2008). The scale was originally developed for patients with chronic pain conditions, but will in the current study be slightly adjusted to apply to patients diagnosed with breast cancer.

Eysenck Personality Questionnaire Revised for Neuroticism (EPQ-R Neuroticism)

The EPQR for Neuroticism is a measure meant to assess neuroticism, defined as a quantitative personality trait believed to predict an individual's vulnerability to neurotic disorders (Birley et al., 2006).There are several versions of the EPQR, each with satisfactory reliability and validity. Usually an EPQR involves other traits as well, but in this study only a 12-item EPQR for neuroticism will be used. Hence, the other traits will be excluded, and only neuroticism will be measured. As with most EPQRs the respondents will be asked to answer either "yes" or "no" on various statements regarding neuroticism and emotional stability (Francis & Pearson, 1988).

Blood analyses

The immune functional assay will consist in collecting whole blood samples using the standardized TruCulture system (Myriad RBM, Austin, Texas, USA) directly containing immunogenic stimuli: medium alone (Null), LPS at 100 ng/mL (from Escherichia coli O55:B5), and Staphylococcus aureus enterotoxin B (SEB) at 0.4 µg/mL, respectively. Immune function is assessed by the commercially available test TruCulture® (Myriad RBM, Austin, USA). TruCulture® reproducibly reveals the induced innate and adaptive immune response in whole blood after stimulation, by quantifying the release of soluble immune activation products (cytokines, chemokines, soluble receptors etc.) in the supernatant and by measuring the transcription level (mRNA) in the circulating blood (immune) cells (53).

Data collection and management

Self-reported data and biomarker data will be collected pre-surgery (questionnaires, hair and blood), post-surgery upon discharge (brief phone survey), 3-4 weeks after surgery (blood), as well as 3 and 6 months after surgery (only questionnaire data). These time points either align with clinical follow-ups at the outpatient oncology department, or are responded to through an

electronic link in the patients' homes to avoid unnecessary strain on the patient. Patient charts will be used to collect data on surgical procedures and potential complications during/after surgery. This will include information about the duration of the surgery, tumor-related variables, severity of acute post-surgical pain, and other relevant intra-operative variables. Registry data on employment and benefit take-up and medical prescriptions will be obtained from FD-Trygd and the Norwegian Prescription Database (NorPD), respectively. To be able to assess for the effects of the interventions, data from NorPD and FD-Trygd will be obtained from inclusion in the trial until one year follow-up. The NorPD contains data about dispensed drugs in Norway, while FD-Trygd contains social security micro data for research. These data will be obtained retrospectively. The post-operative booklet that the patients receive as part of the iACT will also be collected at 6 months follow-up to assess if the patients used it as intended.

------------ INSERT FIGURE 1-----------

Survey data will be collected electronically by the use of laptops/smartphones. We will also utilize data from patients' medical records regarding surgical procedures and medical variables, as well as registry data, which will be linked to the survey data. Data from medical records as well as test results from blood samples will be entered directly into Viedoc by our study nurse. All data will be stored on a secure server at the hospital. The hospital already have a system in place for secure collection and storing of sensitive data which will be used.

A detailed plan for data management and sharing will be developed in collaboration with both Oslo University and the University of Oslo that shares the responsibility for data management in this study.

Implementation

The project will be administered from the Dept of Psychology, University of Oslo, which has the infrastructure to carry out such a study. Data will however be collected, stored and analyzed at Oslo University Hospital through Videoc which is an established and secure system for data collection, storing and management approved by the hospital. The trial will be run at the hospital in the Department of Breast and Endocrine surgery, which is a department with extensive experience in running clinical trials. Two study nurses will be employed to work in the out-patient department and on the ward with patient recruitment and biomarker assessments. A research assistant will be responsible for practical aspects of the trial as well as administering the follow-up data collection which we will attempt to align with the regular medical follow-ups following surgery to ensure a high response rate. Trained therapists and the PhD-student will be responsible for the pre-surgery hypnosis intervention. They will go through didactic and practical training, and complete supervised practice as part of their training. The hypnosis script is already translated and has been pilot tested in a small sample of breast cancer patients (Reme et al *in progress*). The iACT has been developed under the leadership of the co-PI of the study (Jacobsen), with input from our national and international partners (Linton & Flink), and user representatives. The user representatives will further be invited to participate in a focus group upon the completion of the post-operative intervention to discuss their experiences of the intervention and share any advice for modifications or changes to the interventions or procedures.

In order to disseminate the results to patients, clinicians, policy makers and the public, we are planning to co-host a seminar with the Norwegian Cancer Society for stakeholders and

collaborators to present results from the trial. The Cancer Society will also communicate the research findings through their channels, which are well organized and far reaching.

Participants

Number and source of participants

The study will recruit and randomize 200 participants amongst patients from the Department of Breast and Endocrine surgery at Oslo University hospital (see sample size calculations for justification).

Recruitment

Eligible patients will be informed about the study by phone and on their first outpatient appointment at the clinic. A study nurse will be responsible for informing and recruiting patients to the trial.

Inclusion criteria

Patients must fulfill the following criteria to be eligible for inclusion in the study:

- Women diagnosed with breast cancer and scheduled for surgery
- Be able to provide informed consent
- Over the age of 18

Exclusion Criteria

- Insufficient Norwegian speaking or writing skills to participate in the interventions and fill out questionnaires
- Over the age of 80
- cognitive and psychiatric impairment or
- other serious malignancies

Randomization

The randomization procedure will be administered through the program Viedoc that uses an automated and valid randomization procedure. We intend to use a stratified randomization method in order to balance the influence of covariates. The main covariates of interest here is type of surgery (mastectomy vs breast-conserving surgery) and whether the newly diagnosed breast cancer is a relapse or not. Stratified randomization is achieved by generating a separate block for each combination of covariates, and subjects are assigned to the appropriate block of covariates. After all subjects have been identified and assigned into blocks, simple randomization is performed within each block to assign subjects to one of the groups. Furthermore, a SMART randomization procedure will be applied, where the participants are randomized twice – pre-operative (hypnosis vs a one-session mindfulness), and post-operative (iACT vs treatment as usual).

Interventions

Pre-operative hypnosis

The pre-operative intervention involves a brief hypnosis intervention that was previously found effective in reducing acute post-surgical pain and fatigue after breast cancer surgery (41). The hypnosis intervention will replicate a previous study of a 15-minute hypnosis intervention (41), and will be provided in a 15-minute session within 1 hour before surgery. The scripted session includes a relaxation-based induction, suggestions for pleasant visual imagery, suggestions to experience relaxation and peace, and specific symptom-focused suggestions. The intervention will be further adapted and modified to the specific context of this study. Trained therapists will hold the session. Patients will be instructed to use hypnosis

on their own following the intervention session. Based on data from the pilot trial, the Norwegian translation and administration of the hypnosis protocol lasts for 20 minutes.

Active control condition

Patients randomized to the control group will receive a mindfulness based app before surgery. Mindfulness will be delivered via Headspace, a commercially available application which runs on all major smartphones, tablets and web browsers. The Headspace meditation scripts guide users through mindfulness meditations using both audio and visual materials. Internet based mindfulness meditation programs such as Headspace have shown significant effects on chronic pain and fatigue in breast cancer patients (56). However, the referred studies all entail numerous sessions of mindfulness with some degree of therapist involvement. We therefore believe a single session of mindfulness delivered through an app, constitute an active and ethically sound control condition.

Post-surgical intervention

After surgery, participants will be randomized again to either a post-surgical intervention or to treatment as usual. The post-surgical intervention is comprised of a web-based ACT intervention with the cognitive activation model of stress as theoretical vantage points. The intervention consists of videos in combination with a work booklet targeting three main processes derived from the ACT hexaflex model (57), and delivered in two stages. In the initial stage, the intervention has several videos that is built to involve the patient in a broadening of their perspective of what can constitute maintaining and debilitating factors when experiencing persistent pain and/or fatigue. These initial videos involve a biopsychosocial explanation of pain and fatigue, alongside an introduction to mindfulness, values-based living, and stress reduction through cognitive behavioral techniques.

The next stage of the intervention involves the use of a hand-out booklet that serves as an addition to numerous videos presented to the participants. The videos are sorted in three categories aimed to communicate the six processes in a commonsensical, patient friendly way. The three categories are referred to as the tri-flex in the Focused ACT model (58), and consists of Aware (present-moment awareness and self-as-context), Open (Accept and defusion) and Engaged (values and committed action).

Within the booklet there are written tasks corresponding to different videos and scenarios shown in the online intervention to approximate previous trials showing efficacy on chronic pain from a similar intervention (59).

Comparison of interventions

Pre-surgery intervention versus active control: Hypnosis and mindfulness are commonly regarded as supplements or integrative parts of an ACT intervention (43). They both start by focusing the patient's attention in similar ways, but proceed to utilize that focused state into different means. The pre-operative hypnosis here is used with a specific purpose and intent of lessening pain and promote coping. The pre-operative one-session mindfulness has a specific purpose and intent to focus on one thing like the breath, sounds or the body. Thus, both techniques guide the patients experience and use suggestion, but the end goals are fundamentally different.

Drop out and non-compliance

Participants who no longer wish to participate in the study can inform the research group of their decision by notifying the study nurse, the trial coordinator or the PI. The trial

coordinator will in any case contact participants who drop out by phone, and ask if they are willing to report the reason for withdrawal. If reasons are provided, these will be registered on a dedicated drop out form. Whether they only want to withdraw from the interventions, or if they also want to withdraw from future follow-ups, will also be registered. Finally, it will be registered if the participant gives permission for use of data that has already been collected, or if they want these to be deleted. Participants who drop out of the study will still be included in the intention-to-treat (ITT) analyses, unless they required all their data deleted.

Analyses

Sample size and power

Sample size is estimated for both of the primary outcomes. Previous trials with similar interventions have demonstrated moderate/large effect sizes on pain and fatigue (26, 60). However, compared to these studies, we expect more heterogeneity in our data as our study population will undergo several medical treatments during the trial (e.g. radiotherapy or chemotherapy after surgery). We thus expect a minimum difference of one point on NRS (0-10) and 3 points on FACIT-F (which corresponds to patients reporting being "slightly better"). Based on previous studies, it is expected that the control group scores 5 (SD: 2.4) on NRS at 3 months follow-up (25), and 18 on FACIT-F (26). With power set at 0.80 and two-tailed α set at .05, the number needed in each group to detect the change in pain is n=92, and in fatigue n=87. To account for attrition and loss to follow-up, we plan to include 100 patients in each group.

Statistical analyses

Effect analyses of primary and secondary outcomes will be performed according to both the intention-to-treat principle and per protocol. The effect analysis of the primary outcomes will

involve a comparison of pain and fatigue levels at 3 months follow-up. Additionally, they will be analyzed using linear mixed models (also referred to as multilevel models) at the 3 different time points (post-surgery, 3- and 6-months f/u). Multilevel modelling is a flexible statistical approach that can handle non-balanced data with missing entries and repeated observations (61). The health economic analyses will investigate differences in treatment costs, as well as indirect costs related to work (dis)ability and sick-leave. A cost-utility analysis will be performed estimating costs per quality-adjusted life year.

Health Economic analysis

The health economic analyses will investigate differences in treatment costs, as well as indirect costs related to work (dis)ability and sick-leave. The cost-utility analysis will be performed according to well-established methods, taking into account the direct (pre- and post-operative interventions) costs and quality of life (generated from EQ-5D).
performed estimating costs per quality-adjusted life year (QALY).

Compliance

Regular supervision will be conducted with the therapists conducting the pre-operative hypnosis in order to secure compliance to the protocol. The PI and co-PI of the study will be in charge of the supervision. They are both experienced psychologists with a clinical specialization and they are certified in this particular hypnosis intervention. In order to assess compliance with the post-operative intervention, we will have user statistics logged on the web-page i.e. what videos have been used, by which ID and when. This will be used in combination with collecting the work-booklets to see whether the patient has performed different tasks related to the videos. If accepted by GDPR standards, we will associate the

booklet with the given video using an unidentified study ID yielding a rate of completion for the post-operative intervention.

Confidentiality

Participant reported outcome measures (PROMs) will be completed electronically using the ViedocMe functionality available in Viedoc. Study staff will create a ViedocMe account for each participant in the participant's Clinic View in Viedoc and provide an unique log-in profile (user name, pin-code, and ViedocMe web-address) for each participant. Participants are to use this information to log in to their personal ViedocMe account. The questionnaires in ViedocMe will only be available for completion for three days prior to their clinical visitations.

Discussion

Breast cancer is the most common form of cancer among women (1), and a majority of women report persistent psychological and physiological symptoms after breast-cancer surgery (3-5). Chronic post-surgical pain and fatigue are the most common sequelas (6, 11) . This is a both a major clinical problem, with prevalence rates ranging up to 60% (6) , and a socioeconomic burden on the health care system (62). Studies on preventive interventions are scarce, and those that have been carried out have not been sufficiently replicated.

This study will to our knowledge be the first study to investigate the combined effects of a pre-operative hypnosis intervention and a web-based post-operative ACT intervention in women about to undergo breast cancer surgery. It will also be among the very few attempts worldwide to prevent the incidence of pain and fatigue after breast cancer surgery applying a psychological approach. This study design has several strengths. The outcome measures are valid and reliable, involving both self-report measures, objective registry data, and biomarker

data, and the study will contribute to the need of rigorous clinical trials within this field of research. The project results will be highly relevant to all health care personnel involved in breast cancer care, but also to the larger community of clinicians and researchers within the field of chronic pain and fatigue.

Trial Status

The trial will start recruiting in April 2020.

Abbreviations

ACT: Acceptance and Commitment Therapy

CPSP: Chronic Post-Surgical Pain

EQ-5D: European Quality of Life – 5 Dimensions

QoL: Quality of Life

RCT: Randomized Controlled Trials

TAU: Treatment As Usual

Declarations

Acknowledgements

Funding

The study is funded by the Norwegian Cancer Society. The funding sources do not have any role in the design of the study, data collection, analysis and interpretation of data, or decisions to submit articles for publication.

Availability of data and materials

An anonymized dataset will be made available upon request when the main results of the trial are published.

Authors' contributions

SER is the principal investigator of the study, HBJ is the co-PI of the study. SER and HBJ drafted the manuscript and revised it for critical intellectual content.

Competing interests

All authors declare that they have no competing interests.

Consent for publication

Not applicable.

Ethics approval and consent to participate

The study will comply with good clinical practice, including the most recent version of the declaration of Helsinki, as well as all relevant rules and regulations of Norway. The study will be submitted to the Regional ethical committee and the Data Protection Officer at Oslo University Hospital as well as the Data Protection Officer at Oslo University for approval. All participants have signed a signed a statement on consent to participate. The right to withdraw from the study at any time without any explanation, and without any consequences for the further treatment at the clinic, will be emphasized.

Author details

[1] Department of Psychology, Faculty of Social Sciences, University of Oslo, Postboks 1094 Blindern, 0317 Oslo, Norway

[2] Department of Pain Management and Research, Oslo University Hospital, Oslo, Norway

[3] Correspondence: silje.reme@psykologi.uio.no

References

1. McPherson K, Steel CM, Dixon JM. ABC of breast diseases. Breast cancer-epidemiology, risk factors, and genetics. BMJ. 2000;321(7261):624-8.
2. Mouridsen HT, Bjerre KD, Christiansen P, Jensen MB, Moller S. Improvement of prognosis in breast cancer in Denmark 1977-2006, based on the nationwide reporting to the DBCG Registry. Acta Oncol. 2008;47(4):525-36.
3. Gartner R, Jensen MB, Nielsen J, Ewertz M, Kroman N, Kehlet H. Prevalence of and factors associated with persistent pain following breast cancer surgery. JAMA. 2009;302(18):1985-92.
4. Peuckmann V, Ekholm O, Rasmussen NK, Groenvold M, Christiansen P, Moller S, et al. Chronic pain and other sequelae in long-term breast cancer survivors: nationwide survey in Denmark. Eur J Pain. 2009;13(5):478-85.
5. Macdonald L, Bruce J, Scott NW, Smith WC, Chambers WA. Long-term follow-up of breast cancer survivors with post-mastectomy pain syndrome. Br J Cancer. 2005;92(2):225-30.
6. Andersen KG, Kehlet H. Persistent pain after breast cancer treatment: a critical review of risk factors and strategies for prevention. J Pain. 2011;12(7):725-46.
7. Schou Bredal I, Smeby NA, Ottesen S, Warncke T, Schlichting E. Chronic pain in breast cancer survivors: comparison of psychosocial, surgical, and medical characteristics between survivors with and without pain. J Pain Symptom Manage. 2014;48(5):852-62.
8. Mejdahl MK, Andersen KG, Gartner R, Kroman N, Kehlet H. Persistent pain and sensory disturbances after treatment for breast cancer: six year nationwide follow-up study. BMJ. 2013;346:f1865.
9. Boehmke MM, Dickerson SS. Symptom, symptom experiences, and symptom distress encountered by women with breast cancer undergoing current treatment modalities. Cancer Nurs. 2005;28(5):382-9.
10. Huang HP, Chen ML, Liang J, Miaskowski C. Changes in and predictors of severity of fatigue in women with breast cancer: A longitudinal study. Int J Nurs Stud. 2014;51(4):582-92.
11. Bower JE, Ganz PA, Desmond KA, Rowland JH, Meyerowitz BE, Belin TR. Fatigue in breast cancer survivors: occurrence, correlates, and impact on quality of life. J Clin Oncol. 2000;18(4):743-53.
12. Kehlet H, Jensen TS, Woolf CJ. Persistent postsurgical pain: risk factors and prevention. Lancet. 2006;367(9522):1618-25.
13. Macrae WA. Chronic post-surgical pain: 10 years on. Br J Anaesth. 2008;101(1):77-86.
14. Hinrichs-Rocker A, Schulz K, Jarvinen I, Lefering R, Simanski C, Neugebauer EA. Psychosocial predictors and correlates for chronic post-surgical pain (CPSP) - a systematic review. Eur J Pain. 2009;13(7):719-30.
15. Ganz PA, Bower JE. Cancer related fatigue: a focus on breast cancer and Hodgkin's disease survivors. Acta Oncol. 2007;46(4):474-9.
16. Bardwell WA, Ancoli-Israel S. Breast Cancer and Fatigue. Sleep Med Clin. 2008;3(1):61-71.
17. Montgomery GH, Hallquist MN, Schnur JB, David D, Silverstein JH, Bovbjerg DH. Mediators of a brief hypnosis intervention to control side effects in breast surgery patients: response expectancies and emotional distress. J Consult Clin Psychol. 2010;78(1):80-8.
18. VanDenKerkhof EG, Hopman WM, Goldstein DH, Wilson RA, Towheed TE, Lam M, et al. Impact of perioperative pain intensity, pain qualities, and opioid use on chronic pain after surgery: a prospective cohort study. Reg Anesth Pain Med. 2012;37(1):19-27.

19. Wang L, Guyatt GH, Kennedy SA, Romerosa B, Kwon HY, Kaushal A, et al. Predictors of persistent pain after breast cancer surgery: a systematic review and meta-analysis of observational studies. CMAJ. 2016;188(14):E352-E61.
20. De Vries J, Van der Steeg AF, Roukema JA. Determinants of fatigue 6 and 12 months after surgery in women with early-stage breast cancer: a comparison with women with benign breast problems. J Psychosom Res. 2009;66(6):495-502.
21. De Kock M. Expanding our horizons: transition of acute postoperative pain to persistent pain and establishment of chronic postsurgical pain services. Anesthesiology. 2009;111(3):461-3.
22. Meretoja TJ, Andersen KG, Bruce J, Haasio L, Sipila R, Scott NW, et al. Clinical Prediction Model and Tool for Assessing Risk of Persistent Pain After Breast Cancer Surgery. J Clin Oncol. 2017;35(15):1660-7.
23. Pinto PR, McIntyre T, Araujo-Soares V, Costa P, Almeida A. Differential predictors of acute post-surgical pain intensity after abdominal hysterectomy and major joint arthroplasty. Ann Behav Med. 2015;49(3):384-97.
24. Theunissen M, Peters ML, Bruce J, Gramke HF, Marcus MA. Preoperative anxiety and catastrophizing: a systematic review and meta-analysis of the association with chronic postsurgical pain. Clin J Pain. 2012;28(9):819-41.
25. Johannsen M, O'Connor M, O'Toole MS, Jensen AB, Hojris I, Zachariae R. Efficacy of Mindfulness-Based Cognitive Therapy on Late Post-Treatment Pain in Women Treated for Primary Breast Cancer: A Randomized Controlled Trial. J Clin Oncol. 2016;34(28):3390-9.
26. Montgomery GH, D. D, Kangas M, Green S, Sucala M, Bovbjerg DH, et al. Randomized Controlled Trial of a Cognitive-Behavioral Therapy Plus Hypnosis Intervention to Control Fatigue in Patients Undergoing Radiotherapy for Breast Cancer. Journal of Clinical Oncology. 2014;32(6):557-63.
27. Fors EA, Bertheussen GF, Thune I, Juvet LK, Elvsaas IK, Oldervoll L, et al. Psychosocial interventions as part of breast cancer rehabilitation programs? Results from a systematic review. Psychooncology. 2011;20(9):909-18.
28. Johannsen M, Farver I, Beck N, Zachariae R. The efficacy of psychosocial intervention for pain in breast cancer patients and survivors: a systematic review and meta-analysis. Breast Cancer Res Treat. 2013;138(3):675-90.
29. Andersen BL, Farrar WB, Golden-Kreutz DM, Glaser R, Emery CF, Crespin TR, et al. Psychological, behavioral, and immune changes after a psychological intervention: a clinical trial. J Clin Oncol. 2004;22(17):3570-80.
30. Garssen B, Boomsma MF, Meezenbroek Ede J, Porsild T, Berkhof J, Berbee M, et al. Stress management training for breast cancer surgery patients. Psychooncology. 2013;22(3):572-80.
31. Lengacher CA, Johnson-Mallard V, Post-White J, Moscoso MS, Jacobsen PB, Klein TW, et al. Randomized controlled trial of mindfulness-based stress reduction (MBSR) for survivors of breast cancer. Psychooncology. 2009;18(12):1261-72.
32. Lynn SJ, Martin DJ, Frauman DC. Does hypnosis pose special risks for negative effects? A master class commentary. The International journal of clinical and experimental hypnosis. 1996;44(1):7-19.
33. Montgomery GH, David D, Winkel G, Silverstein JH, Bovbjerg DH. The effectiveness of adjunctive hypnosis with surgical patients: a meta-analysis. Anesth Analg. 2002;94(6):1639-45, table of contents.
34. Montgomery GH, DuHamel KN, Redd WH. A meta-analysis of hypnotically induced analgesia: how effective is hypnosis? The International journal of clinical and experimental hypnosis. 2000;48(2):138-53.

35. Redd WH, Montgomery GH, DuHamel KN. Behavioral intervention for cancer treatment side effects. J Natl Cancer Inst. 2001;93(11):810-23.
36. Patterson DR, Hoffman HG, Palacios AG, Jensen MJ. Analgesic effects of posthypnotic suggestions and virtual reality distraction on thermal pain. J Abnorm Psychol. 2006;115(4):834-41.
37. Enqvist B, Bjorklund C, Engman M, Jakobsson J. Preoperative hypnosis reduces postoperative vomiting after surgery of the breasts. A prospective, randomized and blinded study. Acta Anaesthesiol Scand. 1997;41(8):1028-32.
38. Faymonville ME, Fissette J, Mambourg PH, Roediger L, Joris J, Lamy M. Hypnosis as adjunct therapy in conscious sedation for plastic surgery. Reg Anesth. 1995;20(2):145-51.
39. Lang EV, Berbaum KS, Faintuch S, Hatsiopoulou O, Halsey N, Li X, et al. Adjunctive self-hypnotic relaxation for outpatient medical procedures: a prospective randomized trial with women undergoing large core breast biopsy. Pain. 2006;126(1-3):155-64.
40. Spiegel D, Bloom JR. Group therapy and hypnosis reduce metastatic breast carcinoma pain. Psychosom Med. 1983;45(4):333-9.
41. Montgomery GH, Bovbjerg DH, Schnur JB, David D, Goldfarb A, Weltz CR, et al. A randomized clinical trial of a brief hypnosis intervention to control side effects in breast surgery patients. J Natl Cancer Inst. 2007;99(17):1304-12.
42. Ost LG. The efficacy of Acceptance and Commitment Therapy: an updated systematic review and meta-analysis. Behav Res Ther. 2014;61:105-21.
43. McCracken LM, Morley S. The psychological flexibility model: a basis for integration and progress in psychological approaches to chronic pain management. J Pain. 2014;15(3):221-34.
44. Jacobsen HB, Kallestad H, Landro NI, Borchgrevink PC, Stiles TC. Processes in acceptance and commitment therapy and the rehabilitation of chronic fatigue. Scand J Psychol. 2017;58(3):211-20.
45. Ramsey SE, Rounsaville D, Hoskinson R, Park TW, Ames EG, Neirinckx VD, et al. The Need for Psychosocial Interventions to Facilitate the Transition to Extended-Release Naltrexone (XR-NTX) Treatment for Opioid Dependence: A Concise Review of the Literature. Subst Abuse. 2016;10:65-8.
46. Weinrib AZ, Azam MA, Birnie KA, Burns LC, Clarke H, Katz J. The psychology of chronic post-surgical pain: new frontiers in risk factor identification, prevention and management. Br J Pain. 2017;11(4):169-77.
47. Katz J, Weinrib A, Fashler SR, Katznelzon R, Shah BR, Ladak SS, et al. The Toronto General Hospital Transitional Pain Service: development and implementation of a multidisciplinary program to prevent chronic postsurgical pain. Journal of pain research. 2015;8:695-702.
48. Abid Azam M, Weinrib AZ, Montbriand J, Burns LC, McMillan K, Clarke H, et al. Acceptance and Commitment Therapy to manage pain and opioid use after major surgery: Preliminary outcomes from the Toronto General Hospital Transitional Pain Service. Canadian Journal of Pain. 2017;1(1):37-49.
49. Johansen V, Fimland MS. Sluttrapport for prosjektet ved Hysnes Helsefort. Trondheim: NTNU; 2016.
50. Hara KW, Bjorngaard JH, Brage S, Borchgrevink PC, Halsteinli V, Stiles TC, et al. Randomized Controlled Trial of Adding Telephone Follow-Up to an Occupational Rehabilitation Program to Increase Work Participation. J Occup Rehabil. 2017.
51. Yellen SB, Cella DF, Webster K, Blendowski C, Kaplan E. Measuring fatigue and other anemia-related symptoms with the Functional Assessment of Cancer Therapy (FACT) measurement system. J Pain Symptom Manage. 1997;13(2):63-74.

52. Zigmond AS, Snaith RP. The hospital anxiety and depression scale. Acta Psychiatr Scand. 1983;67(6):361-70.
53. Duffy D, Rouilly V, Libri V, Hasan M, Beitz B, David M, et al. Functional analysis via standardized whole-blood stimulation systems defines the boundaries of a healthy immune response to complex stimuli. Immunity. 2014;40(3):436-50.
54. Jensen MP. The validity and reliability of pain measures in adults with cancer. J Pain. 2003;4(1):2-21.
55. Fecho K, Miller NR, Merritt SA, Klauber-Demore N, Hultman CS, Blau WS. Acute and persistent postoperative pain after breast surgery. Pain Med. 2009;10(4):708-15.
56. Veehof MM, Trompetter HR, Bohlmeijer ET, Schreurs KM. Acceptance- and mindfulness-based interventions for the treatment of chronic pain: a meta-analytic review. Cogn Behav Ther. 2016;45(1):5-31.
57. Hayes SC, Luoma JB, Bond FW, Masuda A, Lillis J. Acceptance and commitment therapy: model, processes and outcomes. Behav Res Ther. 2006;44(1):1-25.
58. Strosahl KD, Robinson PJ, Gustavsson T. Brief interventions for radical change: Principles and practice of focused acceptance and commitment therapy: New Harbinger Publications; 2012.
59. Trompetter HR, Bohlmeijer ET, Veehof MM, Schreurs KM. Internet-based guided self-help intervention for chronic pain based on Acceptance and Commitment Therapy: a randomized controlled trial. J Behav Med. 2015;38(1):66-80.
60. Montgomery GH, Kangas M, David D, Hallquist MN, Green S, Bovbjerg DH, et al. Fatigue during breast cancer radiotherapy: an initial randomized study of cognitive-behavioral therapy plus hypnosis. Health Psychol. 2009;28(3):317-22.
61. Jackson DL. Reporting results of latent growth modeling and multilevel modeling analyses: some recommendations for rehabilitation psychology. Rehabilitation psychology. 2010;55(3):272-85.
62. Birch S, Stilling M, Mechlenburg I, Hansen TB. Effectiveness of a physiotherapist delivered cognitive-behavioral patient education for patients who undergoes operation for total knee arthroplasty: a protocol of a randomized controlled trial. BMC musculoskeletal disorders. 2017;18(1):116.
